# Breaking disulfide bonds in a weakly bactericidal α-defensin unleashes a potent antimicrobial peptide with an altered conformation

**DOI:** 10.1371/journal.ppat.1013954

**Published:** 2026-02-09

**Authors:** Gan Luo, Mingzhu Zhao, Qingxia Wang, Yang Zhou, Dan Yao, Jue Zhang, Gang Wang, Junjie Zhang, Chongbing Liao, Wuyuan Lu

**Affiliations:** 1 Shanghai Institute of Infectious Disease and Biosecurity, Key Laboratory of Medical Molecular Virology (MOE/NHC/CAMS), School of Basic Medical Science, Fudan University, Shanghai, China; 2 Key Laboratory of Smart Drug Delivery (MOE), School of Pharmacy, Fudan University, Shanghai, China; Amity University, INDIA

## Abstract

Harnessing antimicrobial peptides as bactericidal agents affords an attractive approach to developing new anti-infective therapies. We found that abolishing disulfide bonding in mouse cryptdin 1 (Crp1), a weakly bactericidal α-defensin of 35 residues, turned it into a potent antimicrobial peptide against Gram-negative bacteria. Here we report that Crp1 in its natively folded β-sheet structure forms high-ordered nanonets to cloak, but not kill, *Escherichia coli*, whereas its disulfide-devoid linear counterpart (L-Crp1) readily disintegrates the bacterial membrane as monomers. L-Crp1 adopts a helix-loop-helix conformation in molecular dynamics simulations, likely conducive to productive peptide-membrane interactions detrimental to bacteria. A truncated peptide spanning the helix-loop-helix, L-Crp1^1-25^, maintains the same conformation as and similar membranolytic and bactericidal activities to L-Crp1. Remarkably, intraperitoneally administered L-Crp1^1-25^ rescues *E. coli*-challenged mice from lethality in a sepsis model by effectively reducing bacterial burden, inflammation and tissue damage. Our studies cultivate additional mechanistic insights into the mode of action of defensins and shed new light on how to harness these host factors for potential therapeutic use.

## Author summary

A recent epidemiological study estimates that antibiotic-resistant bacterial infections directly and indirectly killed 4.71 million patients worldwide in 2021 and will cause 8.22 million annual deaths by 2050. Searching new classes of antibiotics refractory to the existing resistance mechanisms for antibacterial therapy is imperative for global public health and economic growth. Toward this end, cationic antimicrobial peptides are particularly attractive drug candidates as they primarily target the anionic microbial membrane by a mechanism that deters the rise of resistance. However, obstacles abound in their clinical translation, including, but not limited to, subpar bactericidal activity and safety concerns. In studying a weakly bactericidal mouse α-defensin, cryptdin 1 (Crp1), we made a surprising finding that breaking its three structurally stabilizing disulfide bonds through Cys-to-Ala mutations unleashed an antimicrobial peptide against Gram-negative bacteria with an altered conformation but significantly improved potency and selectivity. A further shortened peptide of 25 amino acid residues, L-Crp1^1-25^, was safe and highly efficacious in rescuing *E. coli*-infected mice from lethality by reducing bacterial burden, inflammation and tissue damage. Our study demonstrates how to harness naturally occurring antimicrobial peptides as novel anti-infective agents to combat increasingly prevalent antibiotic resistance.

## Introduction

Antimicrobial peptides, first discovered by Han G. Boman and colleagues from *Hyalophora cecropia* in 1981 [1], are ubiquitously present in multicellular organisms as diverse as humans, amphibians, insects, plants, and protozoa, constituting the first line of host defense against microbial infection [2]. A major class of antimicrobial peptides found in mammals are defensins [3–5], which were first identified by Robert I. Lehrer and colleagues from rabbit macrophages and human neutrophils in the 1980’s [6–9]. These cationic and Cys-rich peptides of 2–5 kDa are classified into three structural families based on the topology of three intramolecular disulfide bonds, i.e., α-, β-, and θ-defensins [3,10], and play important roles in innate immunity against bacterial, viral and fungal pathogens [11,12].

α-Defensins feature a disulfide topology of Cys1-Cys6, Cys2-Cys4 and Cys3-Cys5, while a Cys1-Cys5, Cys2-Cys4 and Cys3-Cys6 connectivity is characteristic of β-defensins. Despite the difference in disulfide bonding, both α- and β-defensins adopt a highly similar, three-stranded β-sheet tertiary structure [5,13]. By contrast, θ-defensins of 18 amino acids residues, which are found only in primates but not in humans due to a premature stop codon, are head-to-tail cyclized with three disulfides arranged in a ladder pattern [14,15]. For humans, there are only six α-defensins, including four human neutrophil peptides (HNP1-4) and two human defensins (HD5-6) expressed by Paneth cells in the small intestine [16], and dozens of β-defensins found primarily in epithelial cells and tissues [17]. Interestingly, mice do not express α-defensins in neutrophils [18]. They do, however, express many α-defensins in the crypts of the small intestine, termed cryptdins [19], along with scores of mouse β-defensins in the epithelia of various organs [20]. Of note, disulfide bonding is often required for many host protective and immunomodulatory functions of defensins where a stable tertiary or quaternary structure necessitates their productive interactions with various proteinaceous targets such as microbial proteins and host receptors [21–24]. However, the functional importance of disulfide bonding for the bactericidal activity of defensins is far from certain.

Defensins kill bacteria through two major mechanisms, i.e., disruption of the plasma membrane of Gram-negative bacteria [25–27] and inhibition of the cell wall synthesis of Gram-positive bacteria [28,29]. Significant progress has been made in advancing our understanding of the molecular basis (cationicity, hydrophobicity, disulfide bonding, quaternary structure, etc.) that underlies defensin functions [5]. Disulfide bonding in the human α-defensins HNP1 and HD5, for example, while often dispensable for the killing of Gram-negative bacteria, can be critical for killing Gram-positive bacteria [30,31]. However, how disulfide bonding in a particular defensin influences its antimicrobial activity against a specific pathogen remains difficult to predict and poorly understood.

In fact, loss of the three disulfide bonds in defensins has been reported to enhance, reduce, or have little effect on their bactericidal activity compared with their natively folded forms [30–36]. In studying three mouse enteric α-defensins, cryptdin 1 (Crp1), cryptdin 4 (Crp4) and cryptdin 14 (Crp14), we found that the contribution of the three disulfide bonds to cryptdin activity was sequence-dependent and bacterial species-specific [36], confounding the mechanistic complexity of these peptides from the same structural family. Crp1 is active against *Staphylococcus aureus*, while its disulfide-devoid form kills *S. aureus* with modestly reduced activity [36]. Remarkably, loss of disulfide bonding in Crp1, which, itself, is among the weakest of all 17 cryptdins studied against *Escherichia coli*, results in a linear Crp1 peptide with the strongest *E. coli*-killing activity in the panel [36]. In sharp contrast, loss of disulfide bonding in human neutrophil peptide 4 (HNP4) substantially reduces its bactericidal activity against both *S. aureus* and *E. coli* [35]. This report aims to address the mechanistically important questions of why and how a linearized defensin peptide exhibits a drastically different bactericidal activity against Gram-negative bacteria compared with its natively folded parent molecule. Efforts to answer these questions have led to the design and mechanistic elucidation of a shortened linear peptide derived from Crp1 with significant *in vitro* and *in vivo* activities against *E. coli* infection.

## Results

### Comparison of the primary, tertiary and quaternary structures of Crp1, Crp14 and HNP4

**Fig 1:**
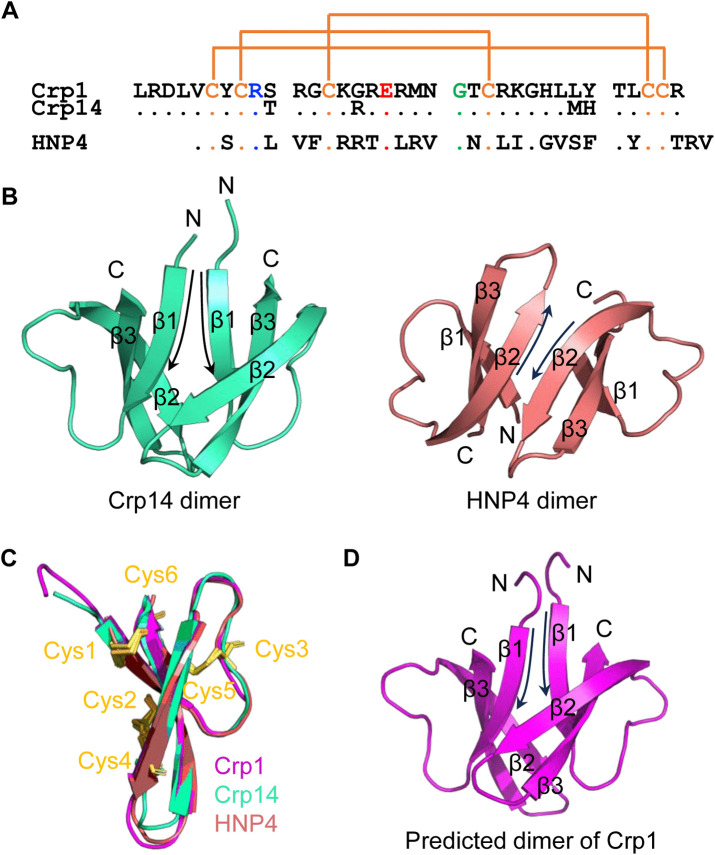
Structural comparison of Crp1, Crp14 and HNP4. (A) Amino acid sequences of Crp1, Crp14 and HNP4, with conserved residues highlighted by colors (Cys in orange, Arg in blue, Glu in red, and Gly in green) and disulfide bonds by orange lines. (B) Different dimerization modes of Crp14 and HNP4. Crp14 forms a unique noncanonical dimer (cyan, PDB: 7YOA) via parallel β1-β1 interactions, while HNP4 (red, PDB: 1ZMM), like other mammalian α-defensins, form a dimer through antiparallel β1-β1 interactions. (C) Structural superposition of the monomers of Crp1 (purple, predicted by AlphaFold), Crp14 (cyan, PBD: 7YOA) and HNP4 (red, PDB: 1ZMM). (D) The predicted dimer of Crp1 exhibits a noncanonical mode similar to Crp14.

The amino acid sequence of Crp1 of 35 residues is as follows: LRDLVCYCR**S**^10^ RGCK**G**RERMN^20^ GTCRKGHL**LY**^30^ TLCCR^35^ (Fig 1A). It contains 6 Cys residues, 9 cationic residues, 2 anionic residues, 9 hydrophobic residues, 5 hydrophilic residues, and 4 Gly residues. Crp14 (LRDLVCYCR**T**^10^ RGCK**R**RERMN^20^ GTCRKGHL**MH**^30^ TLCCR^35^) differs from Crp1 by four mutations [37], i.e., S10T, G15R, L29M and Y30H (Fig 1A). We previously solved the crystal structure of Crp14 [36] (Fig 1B), which adopts a disulfide-stabilized three-stranded β-sheet conformation as seen in all mammalian α-defensins with known structures [38–41]. However, Crp14 is unique in that it forms a noncanonical dimer through asymmetrical β1-β1 interactions in parallel (Fig 1B). This mode of dimerization differs from that of other mammalian α-defensins such as HNP4 (Fig 1B), where antiparallel β2-β2 interactions mediate dimer formation [38].

To decipher structural characteristics of Crp1, we conducted an unbiased prediction of its three-dimensional model by AlphaFold (Fig 1C). The predicted structure of Crp1 displayed a tertiary fold nearly identical to that of Crp14 or HNP1, with a three-stranded β-sheet stabilized by three disulfide bridges (Fig 1C). Given high sequence identity, a model of Crp1 dimer was constructed and fully relaxed by Rosetta according to the crystal structure of Crp14, yielding, as expected, the non-canonical mode of dimerization seen in Crp14 (Fig 1D).

For this study, we replaced all six Cys residues in Crp1 with an Ala residue, resulting in an unstructured linear peptide termed L-Crp1 (S1 Fig). As controls, HNP4 (VCSCRLVFCR^10^ RTELRVGNCL^20^ IGGVSFTYCC^30^ TRV) and its Ala-substituted linear form L-HNP4 were also included (Fig 1A) [35].

### Linearization markedly enhances the bactericidal activity of Crp1 against Gram-negative bacteria

**Fig 2:**
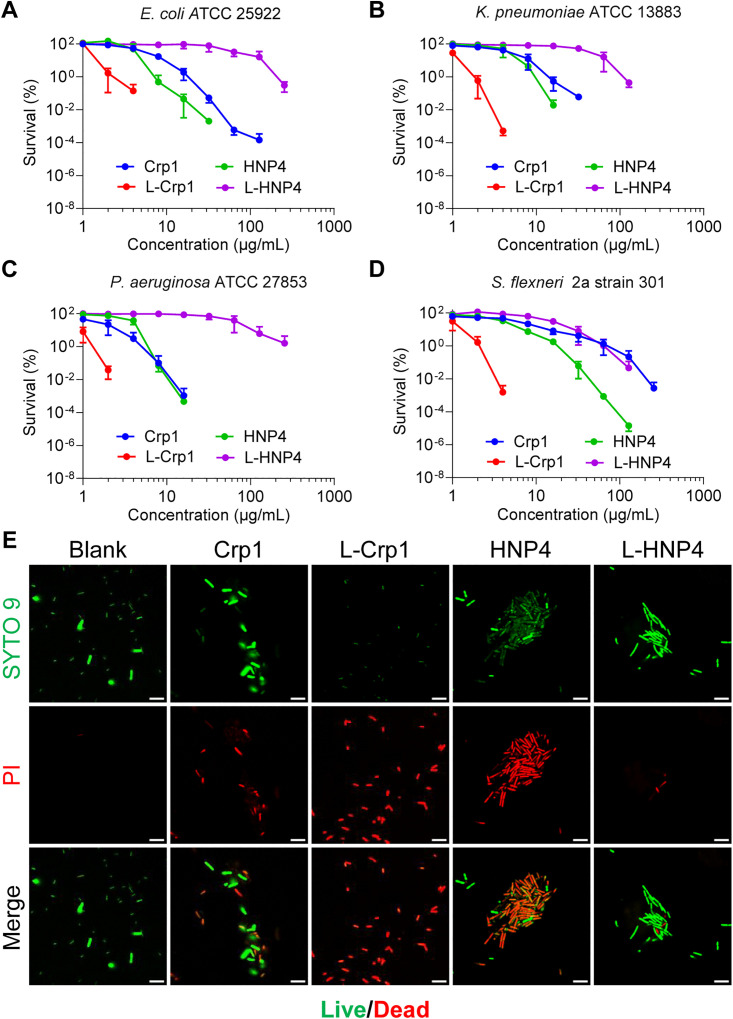
Linearization of Crp1 significantly enhances its bactericidal activity against Gram-negative bacteria. (A-D) The bactericidal activities of the indicated peptides on *E. coli* ATCC 25922 (A), *K. pneumoniae* ATCC 13883 (B), *P. aeruginosa* ATCC 27853 (C) and *S. flexneri* 2a strain 301 (D). The bacterial survival was quantified using the virtual colony count assay. Results are mean ± SD of at least three independent experiments. (E) Confocal microscopy images of *E. coli* ATCC 25922 treated by 4 μg/ml of the indicated peptides for 1 hour. The untreated bacteria were used as controls (Blank). The live and dead bacteria were stained by SYTO 9 (green) and PI (red), respectively. Scale bar = 5 µm.

To verify the separately reported findings on Crp1, L-Crp1, HNP4 and L-HNP4 with *E. coli* [35,36], we subjected the four defensin peptides to the virtual colony count (vCC) assay [42], where a serially diluted peptide (from 256 to 1 μg/ml) in 10 mM sodium phosphate buffer, pH 7.4, was incubated at 37 °C with bacteria (1 × 10^6^ CFU/ml) for 2 h, followed by a 12-hour spectrophotometric monitoring of bacterial growth in Mueller-Hinton broth. The vCC assay significantly simplifies the procedures of labor-intensive, actual colony counting assays, and is presumably more accurate than traditional broth microdilution methods as designed [42]. The defensin dose-dependent bacterial survival curves of *E. coli* ATCC 25922, *K. pneumoniae* ATCC 13883, *P. aeruginosa* ATCC 27853, and *S. flexneri* 2a strain 301 are shown in Fig 2A-D; the peptide concentrations, at which 50%, 90%, 99% and 99.9% of input bacteria were killed, are reported as virtual lethal dose 50 (vLD50), vLD90, vLD99 and vLD99.9, respectively, and tabulated in Table 1.

**Table 1:** Antibacterial virtual lethal doses of Crp1, L-Crp1, HNP4, L-HNP4, L-Crp1^1-14^, L-Crp1^1-25^ and polymyxin B (PMB). The virtual lethal doses (vLD) of peptides required to kill 50%, 90%, 99%, and 99.9% of viable cells from various gram-negative bacteria inputs were determined. These data were derived from three independent assays, and assays for each peptide were performed together.

Bacteria	Peptides	vLD50	vLD90	vLD99	vLD99.9
***E. coli*** **ATCC 25922**	Crp1	3.93 ± 0.71	9.45 ± 0.56	17.66 ± 2.00	27.81 ± 2.55
	L-Crp1	1.12 ± 0.02	1.46 ± 0.13	2.01 ± 0.29	>4
	HNP4	3.45 ± 1.00	4.31 ± 1.28	7.24 ± 1.36	12.00 ± 3.95
	L-HNP4	74.31 ± 21.75	128.17 ± 41.04	175.63 ± 65.96	>256
	L-Crp1^1–14^	63.74 ± 16.31	91.30 ± 24.44	115.31 ± 28.22	143.23 ± 30.55
	L-Crp1^1–25^	2.09 ± 0.41	2.95 ± 0.36	4.03 ± 0.55	5.21 ± 0.51
	PMB	0.51 ± 0.05	1.18 ± 0.24	1.46 ± 0.10	>2
***K. pneumoniae*** **ATCC 13883**	Crp1	2.34 ± 0.58	5.63 ± 0.02	10.56 ± 0.08	20.23 ± 5.45
	L-Crp1	<1	1.20 ± 0.09	1.76 ± 0.26	2.27 ± 0.20
	HNP4	3.54 ± 1.49	6.11 ± 2.06	8.58 ± 2.62	11.85 ± 2.92
	L-HNP4	33.67 ± 2.64	64.42 ± 13.88	107.63 ± 2.39	>256
	L-Crp1^1–14^	30.75 ± 2.36	45.55 ± 2.03	>256	>256
	L-Crp1^1–25^	1.14 ± 0.04	1.95 ± 0.14	4.18 ± 0.27	5.83 ± 0.23
	PMB	0.07 ± 0.02	0.76 ± 0.32	>1	>1
***P. aeruginosa*** **ATCC 27853**	Crp1	1.57 ± 0.95	2.48 ± 0.95	3.32 ± 0.63	6.54 ± 2.51
	L-Crp1	<1	1.02 ± 0.02	1.27 ± 0.14	1.72 ± 0.15
	HNP4	3.22 ± 0.82	4.57 ± 0.17	5.91 ± 0.15	7.64 ± 0.51
	L-HNP4	52.96 ± 24.90	100.55 ± 65.76	>256	>256
	L-Crp1^1–14^	11.56 ± 3.31	29.20 ± 12.97	33.53 ± 6.90	39.79 ± 1.35
	L-Crp1^1–25^	1.03 ± 0.02	1.29 ± 0.19	1.85 ± 0.61	1.97 ± 0.62
	PMB	0.07 ± 0.01	0.88 ± 0.21	>2	>2
***S. flexneri*** **2a strain 301**	Crp1	2.08 ± 1.12	13.90 ± 3.31	56.98 ± 26.66	121.38 ± 54.53
	L-Crp1	<1	1.36 ± 0.21	1.88 ± 0.34	2.38 ± 0.39
	HNP4	2.59 ± 0.31	10.97 ± 4.29	17.95 ± 1.50	27.91 ± 5.59
	L-HNP4	9.93 ± 0.17	28.66 ± 8.86	55.64 ± 27.61	86.02 ± 56.24
	L-Crp1^1–14^	18.13 ± 0.30	26.65 ± 1.44	34.94 ± 0.73	39.91 ± 1.04
	L-Crp1^1–25^	1.92 ± 0.26	2.43 ± 0.14	3.11 ± 0.20	3.97 ± 0.28
	PMB	0.15 ± 0.09	0.54 ± 0.26	1.15 ± 0.02	1.37 ± 0.06

As shown in Fig 2A and Table 1, the order of bactericidal activity of the four peptides against *E. coli* is: L-Crp1 > HNP4 > Crp1 > L-HNP4. L-Crp1 completely eradicated *E. coli* growth at 8 μg/ml, whereas Crp1 achieved the same level of activity at 256 μg/ml. L-Crp1 reduced *E. coli* growth by 2 orders of magnitude at 2.01 μg/ml (vLD99), a concentration 9-fold lower than that required of Crp1 (17.66 μg/ml). By contrast, L-HNP4 was significantly weaker than HNP4 as the vLD ratios of L-HNP4 to HNP4 generally fell between 20 and 30. In fact, while HNP4 killed off *E. coli* at 64 μg/ml, L-HNP4 at 256 μg/ml reduced *E. coli* growth by only ~2.5 logs.

Similar observations were made with three additional Gram-negative bacteria, *K. pneumoniae*, *P. aeruginosa*, and *S. flexneri*. As shown in Fig 2B-D and Table 1, L-Crp1 and HNP4 were significantly more potent than Crp1 and L-HNP4, respectively, consistent with their activity profiles on *E. coli*. As the most active peptide in the panel, L-Crp1 quantitatively killed *K. pneumoniae* at 8 μg/ml and *P. aeruginosa* at 4 μg/ml, roughly one order of magnitude more potent than Crp1. L-Crp1 was significantly more active than Crp1 against *S. flexneri*, registering a 30–50-fold difference in vLD99 and vLD99.9 values. Overall, the order of susceptibility of the bacteria to the killing by L-Crp1/Crp1 appears: *P. aeruginosa* > *K. pneumoniae* > *E. coli* > *S. flexneri*. We also verified the killing of *E. coli* by each peptide at 4 μg/ml using live/dead cell staining and confocal laser scanning microscopy (Fig 2E). Collectively, these results are in both qualitative and quantitative agreement with the published reports on Crp1/L-Crp1 and HNP4/L-HNP4 [35,36], that is, loss of disulfide bonding, while greatly reducing the bactericidal activity of HNP4, markedly enhances Crp1 killing of Gram-negative bacteria.

### Linearization substantially augments the membranolytic activity of Crp1

**Fig 3:**
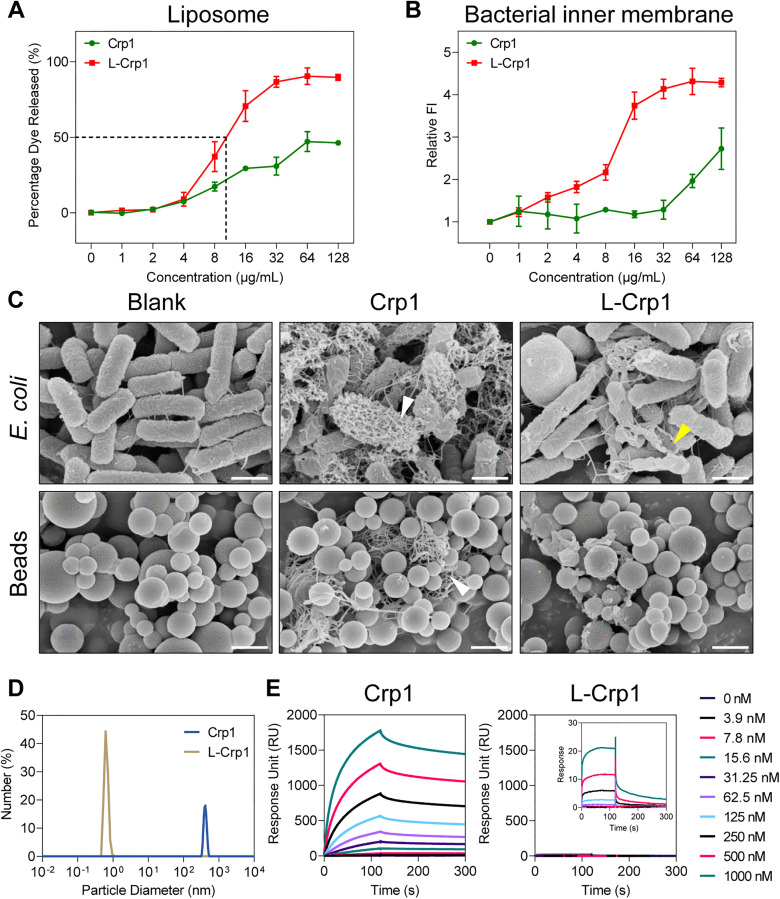
Linearization enhances the membranolytic activity of Crp1, whereas high-order structural assembly reduces its bactericidal efficacy. (A) The LUV membrane disruption by Crp1 and L-Crp1 detected by the fluorescent dye release. The leakage level is presented as the percentage of the dye released after a two-hour incubation. Data are mean ± SD from triplicate assays. (B) Inner membrane permeabilization of *E. coli* ATCC 25922 treated by Crp1 and L-Crp1 was quantified by PI staining. Increased fluorescence intensity (FI) indicates enhanced membrane permeability. Data are mean ± SD of two biological replicates. (C) SEM images of *E. coli* ATCC 25922 treated by Crp1 and L-Crp1 for 2 hours. Protein A polystyrene beads (Beads) and untreated groups (Blank) were used as controls. Nanonet structures (white arrows) existed in the Crp1-treated groups, while membrane disruption and bacterial shrinkage were observed in the L-Crp1-treated bacteria (yellow arrow). Scale bar = 1 μm. (D) Comparison of particle size distributions between Crp1 and L-Crp1 by DLS analysis. (E) Self-association kinetics of Crp1 and L-Crp1 determined by SPR.

It is generally accepted that cationic antimicrobial peptides, regardless of their structural classes, kill Gram-negative bacteria primarily through membrane disruption [2–5,43–45]. Phospholipids-comprised large unilamellar vesicles (LUVs) are often used as a model membrane system to quantify the membranolytic activity of AMPs for mechanistic studies [28,46,47]. We used a previously developed LUV system made of palmitoyl-oleoyl-phosphatidylcholine (POPC) and palmitoyl-oleoyl-phosphatidylglycerol (POPG) at 1:1 molar ratio to encapsulate 8-aminonaphthalene1,3,6-trisulfonic acid sodium salt (ANTS) and p-xylene-bis-pyridinium bromide (DPX) [28,47]. The fluorescent dye ANTS remains quenched by DPX when sequestered together in the liposome and emits fluorescence upon membrane rupture and release into the bulk (λ_ex_ = 350 nm, λ_em_ = 420 nm). As shown in **Fig 3A**, peptide-induced membrane disruption as measured by changes in ANTS fluorescence or percent dye release progressively increased as L-Crp1 or Crp1 concentration was raised in a 2-fold dilution series from 0 to 128 μg/ml. L-Crp1 at ~12 μg/ml induced 50% release of ANTS, whereas the percent release maximized at ~50% even at the highest concentration of 128 μg/ml of Crp1. Similar results were obtained in an *E. coli* inner membrane permeability assay using propidium iodide (PI) as probe. PI enters bacterial cells only when the inner membrane is damaged and emits red fluorescence upon binding to intracellular nucleic acids [48]. As shown in **Fig 3B**, L-Crp1 caused dose-dependent membrane leakage far more efficiently than Crp1 did. Notably, the maximal disparity in membranolytic activity between L-Crp1 and Crp1 arrived at the same concentration of 32 μg/ml in both assays (Fig 3A-B). Overall, the membranolytic activities of L-Crp1 and Crp1 are in accord with their bactericidal activities against Gram-negative bacteria, indicative of membrane disruption as the overriding lethal event leading to bacterial killing by these peptides.

### Linearization unravels the high-order structure of Crp1 detrimental to bacterial killing

To gain additional insight into the functional supremacy of L-Crp1 over Crp1 with respect to its bactericidal and membranolytic activity, we incubated L-Crp1 or Crp1 at 6 μg/ml with *E. coli* ATCC 25922 (~1 × 10^8^ CFU/ml) for 2 hours at 37°C, followed by scanning electron microscopy (SEM) analysis. As controls, protein A-coated polystyrene particles (0.1% w/v) were treated with L-Crp1 or Crp1 under identical conditions. As shown in Fig 3C, Crp1, in sharp contrast with L-Crp1, formed high-order nanonet structures surrounding bacterial cells, whereas L-Crp1 punched visible holes in the *E. coli* membrane, caused shrinkage of the bacterium, and induced bacteriolysis. Crp1 nanonets were also found around protein Α beads, but not with L-Crp1 (Fig 3C), indicating that the nanonets were not of bacterial origin. The strong tendency for Crp1 to oligomerize/multimerize would significantly reduce its effective concentration against *E. coli*, thereby explaining at least in part its inferior bactericidal and membranolytic activities to those of L-Crp1. Consistently, this phenomenon contrasts the HNP4/L-HNP4 pair, where the less active L-HNP4 peptide indeed showed a stronger tendency than HNP4 to form nanonets not only on *E. coli* but also on protein A beads (S2 Fig).

For further verification, we analyzed L-Crp1 and Crp1 at 30 μM each in 10 mM phosphate buffer (pH 7.4), using dynamic light scattering (DLS) techniques, and confirmed the existence of monomeric L-Crp1 (0.5 to 1 nm in particle size) and multimeric Crp1 (300–500 nm in particle size) (Fig 3D). Additionally, HNP4 was observed to be monomeric, while L-HNP4 exhibited as multimeric (S3 Fig). We also immobilized L-Crp1 or Crp1 on CM5 sensor chips (969.6 and 1839.2 RUs, respectively) and followed their self-association kinetics at 25 °C in 10 mM HEPES buffer containing 150 mM NaCl, 3 mM EDTA and 0.005% surfactant P20, pH 7.4, using surface plasmon resonance (SPR) techniques. As shown in Fig 3E, while Crp1 strongly self-associated in a dose-dependent manner (with very slow dissociation kinetics), L-Crp1 showed a drastically reduced tendency to self-associate (with very fast dissociation kinetics). Collectively, these results support the published finding that the bactericidal activity of cryptdins against *E. coli* is negatively correlated with their ability to self-associate in solution [36].

### L-Crp1 adopts an α-helical conformation conducive to productive peptide-membrane interactions

**Fig 4:**
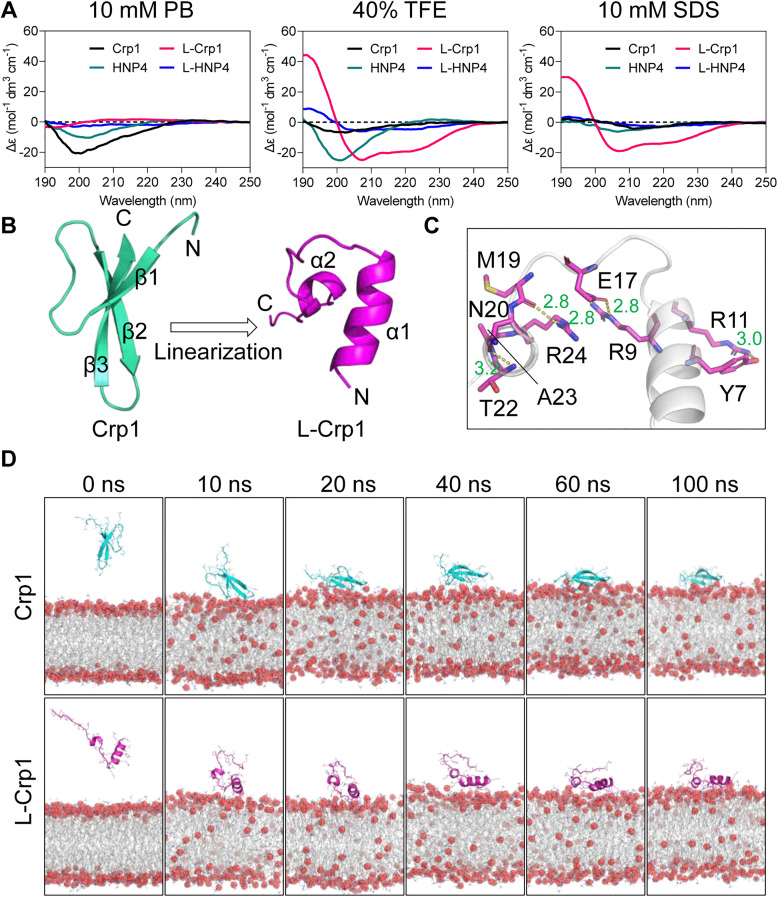
L-Crp1 forms an α-helical conformation that facilitates its interaction with the bacterial membrane. (A) CD spectroscopy of the indicated peptides in various solutions. The spectra were measured from 190 to 250 nm at room temperature with 30 μM of each peptide. (B) The double α-helical structure of L-Crp1 obtained by MD simulations following Crp1 linearization. (C) The local interactions contributing the helix-loop-helix conformation of L-Crp1. (D) MD simulation snapshots of Crp1 and L-Crp1 binding to the bacterial membrane at different time points (0 ~ 100 ns).

Since membrane disruption is an obligate step in the killing of *E. coli*, how productive the interaction is between a defensin peptide and the microbial membrane necessarily dictates its bactericidal activity. To elucidate the mechanisms of action of L-Crp1, Crp1, L-HNP4 and HNP4, we analyzed their conformations using circular dichroism spectroscopy in 10 mM sodium phosphate buffer, pH 7.4, 40% trifluoroethanol (TFE) or 10 mM sodium dodecyl sulphate (SDS). As shown in Fig 4A, these peptides in 10 mM sodium phosphate buffer existed either in a β-sheet conformation (negative peaks at 205–210 nm) as expected for Crp1 and HNP4 [36,38], or in random coils as expected for L-Crp1 and L-HNP4. However, upon addition of the helix-inducing agent TFE [49], L-Crp1, but not L-HNP4, adopted an α-helical conformation (a positive peak at 195 nm and double negative peaks at 208 nm and 222 nm). Importantly, the α-helicity of L-Crp1 was maintained in the presence of the membrane-mimicking agent SDS [50], indicating that L-Crp1 has an intrinsically strong propensity to adopt an α-helical conformation both in TFE-containing solution and in the membrane environment.

Molecular dynamics (MD) simulations of L-Crp1 indeed confirmed the presence of a global double α-helix structure in 10 mM NaCl (Fig 4B), with the first helix spanning amino acid residues 2–12 and the second helix encompassing amino acid residues 20–25, connected by a flexible loop composed of residues 13–19. Locally, an H-bond formed between the side chains of Y7 and R11 stabilizes the first helix, and the second helix is rigidified by an M19 O-R24 N^^ζ^2^ H-bond, two pairs of H-bonds donated by T22 and A23 N to N20 O^γ^, and a salt bridge between R9 and E17 (Fig 4C). These local interactions collectively contribute to the formation of the helix-loop-helix conformation of L-Crp1 in MD ensembles. This helix-loop-helix structure is reminiscent of the helix-hinge-helix pattern observed in cecropin A, the first known antimicrobial peptide discovered by Han G. Boman and colleagues from *Hyalophora cecropia* with a broad-spectrum bactericidal activity against both Gram-negative and Gram-positive bacteria [1,51,52]. In the absence of a more definitive free or membrane-bound structure of L-Crp1 determined by X-ray crystallography or NMR spectroscopy, we hypothesized that productive peptide-membrane interactions ensued from this predicted or calculated double-helix structure of L-Crp1, thus functionally relevant to its potent bactericidal activity against *E. coli*.

**Fig 5:**
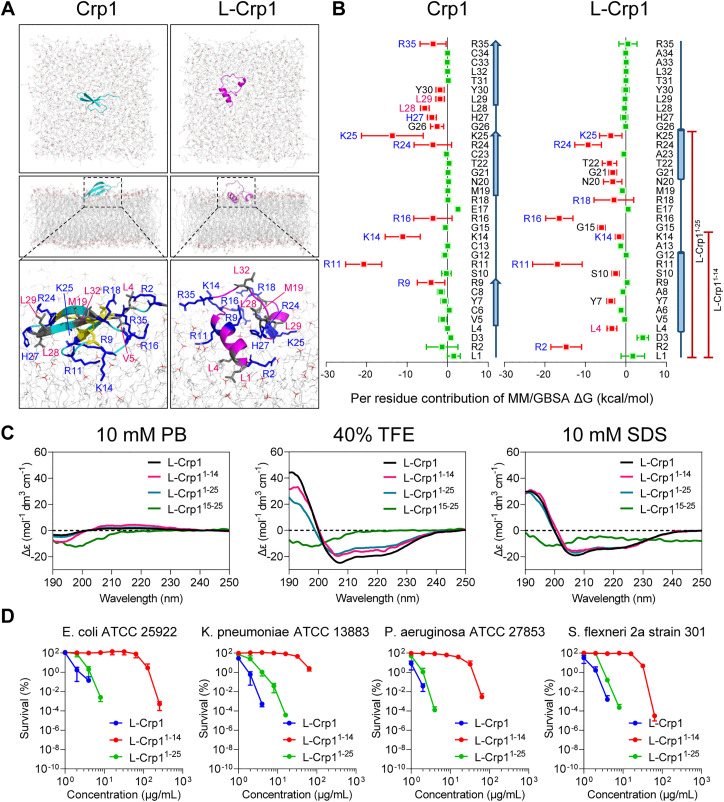
The N-terminal residues 1-25 constitute the key functional domain of L-Crp1. (A) Final poses of the two MD simulation trajectories showing the interactions of Crp1 (blue) and L-Crp1 (red) with the phospholipid bilayer membrane. (B) Per residue energetic contributions of Crp1 and L-Crp1 in membrane binding, calculated by MM/GBSA ΔG decomposition. (C) CD spectra of L-Crp1 and its truncated analogs (L-Crp1^1-14^, L-Crp1^1-25^ and L-Crp1^15-25^), each at 30 μM, measured in various solutions from 190 to 250 nm at room temperature. For comparison, the spectrum of L-Crp1 was taken from Fig 4A. (D) The bactericidal activities of L-Crp1 and its truncated analogs against the indicated bacteria, which were determined along with the peptides described in Fig 2A-D. Results are mean ± SD of at least three independent experiments.

To verify, we visualized the binding of the inner membrane of Gram-negative bacteria with Crp1 and L-Crp1, by employing MD simulations. As illustrated in Fig 4D, both Crp1 and L-Crp1 approached the bacterial membrane within 10 ns and bound firmly until 100-ns production simulation, implicating membrane perturbation as a probable mechanism of action of the two peptides. To map out the interactive details, we placed the two peptides on the outer leaflet of the membrane with the same center-of-mass distances, followed by an additional 200-ns simulation. Poses of the last frames of the two trajectories distinctly illustrated a deeper penetration of L-Crp1 into the membrane than that of Crp1, allowing the two α-helices to present extensively exposed basic residues for close interactions with the membrane (Fig 5A). By contrast, Arg residues in Crp1, which is locked in a stable structure by three disulfide bridges, had limited exposure for productive contacts with the membrane (Fig 5A), likely attributing to its weakly bactericidal activity. Calculations of MM/GBSA ΔG further supported the above analysis, as the binding energy of L-Crp1 (-167.31 ± 18.58 kcal/mol) to engage the membrane was significantly stronger than that of Crp1 (-125.17 ± 29.67 kcal/mol). These MM/GBSA ΔG values were further decomposed into per residue energetic contributions for both peptides (Fig 5B), lending additional support to the two distinct modes of action, where residues from Crp1 and L-Crp1 differentially contributed to their membrane binding.

Of note, the disordered C-terminal segment of L-Crp1, which comprises amino acid residues 26–35, was not engaged by the membrane for interactions as evidenced by MD simulations (Fig 5B), thus functionally dispensable. To validate the MD findings, we synthesized three truncated peptides derived from L-Crp1 for functional characterization, L-Crp1^1-14^ encompassing the α1 helix, L-Crp1^1-25^ encompassing α1/ α2 helices, and L-Crp1^15-25^ encompassing the α2 helix.

### Only L-Crp1^1-25^ functions similarly to L-Crp1

We analyzed the conformations of L-Crp1^1-14^, L-Crp1^1-25^ and L-Crp1^15-25^ in solution by CD spectroscopy and, as shown in Fig 5C, L-Crp1^1-14^ and L-Crp1^1-25^, but not L-Crp1^15-25^, adopted random coils in aqueous buffer at neutral pH and became helical in the presence of either 40% TFE or 10 mM SDS. Not surprisingly, the hydrophilic L-Crp1^15-25^ peptide, despite its three net positive charges, failed to show any bactericidal activity at up to 256 μg/ml against *E. coli* and *S. aureus* (S2 Table), underscoring the importance of a combination of α-helicity and hydrophobicity, in addition to cationicity, for microbial membrane disruption. The bactericidal activity of L-Crp1^1-14^ and L-Crp1^1-25^ peptides against the four Gram-negative bacteria was quantified in a vCC assay (Fig 5D and Table 1). L-Crp1^1-25^ was marginally less active than L-Crp1 with respect to bacterial killing, whereas L-Crp1^1-14^ was substantially weaker than either L-Crp1^1-25^ or the full-length linear peptide control. The vLD values of L-Crp1^1-25^ for *E. coli*, *K. pneumoniae* and *S. flexneri* were higher by a factor of 2 than, and similar for *P. aeruginosa* to, those of L-Crp1, whereas the vLD values of L-Crp1^1-14^ were 1–2 orders of magnitude higher. The disparity in bactericidal activity between the two truncated peptides was not surprising as the 2^nd^ helix with a net positive charge of +3 was shown to be critical for interactions with the microbial membrane (Fig 4C-D).

**Fig 6:**
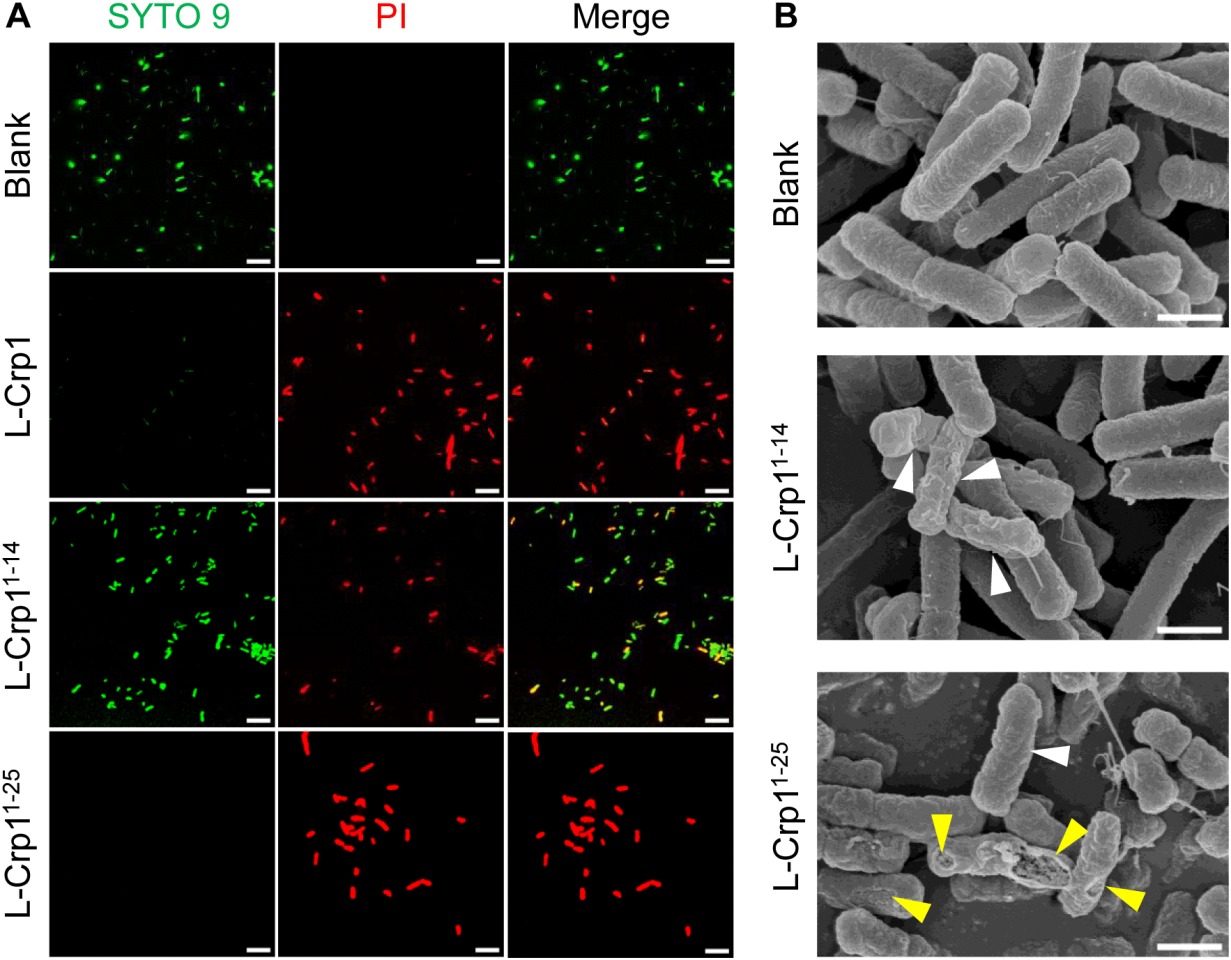
The truncated L-Crp1^1-25^ exhibits comparable bactericidal activity to the full-length L-Crp1. (A) Confocal microscopic images of *E. coli* ATCC 25922 treated with 4 μg/ml of L-Crp1 and its truncated analogs for 1 hour. Untreated bacteria were used as controls (Blank). The live and dead bacteria were stained by SYTO 9 (green) and PI (red), respectively. Scale bar = 5 µm. (B) SEM images of *E. coli* ATCC 25922 treated with truncated analogs of L-Crp1 for 2 hours, which were collected together with the peptides described in S2 Fig. Peptide-punctured holes in the membrane were labelled by yellow arrows, and shrinking bacteria depicted by white arrows. Scale bar = 1 μm.

To further verify bacterial killing by L-Crp1^1-14^ and L-Crp1^1-25^, *E. coli* ATCC 25922 (1 × 10^7^ CFU/ml) was incubated with each peptide at 4 μg/ml for 1 hour at 37°C and analyzed by confocal laser scanning microscopy. As shown in Fig 6A, live/dead cell staining clearly depicted L-Crp1^1-25^ superior to L-Crp1^1-14^ in the killing of *E. coli*. SEM analysis showed that neither peptide formed high-order structures surrounding dying *E. coli* and membrane disruption appeared to be responsible for the demise of the bacteria (Fig 6B), thus consistent with the mode of action of L-Crp1.

### L-Crp1^1-25^ rescues mice challenged with *E. coli*

**Fig 7:**
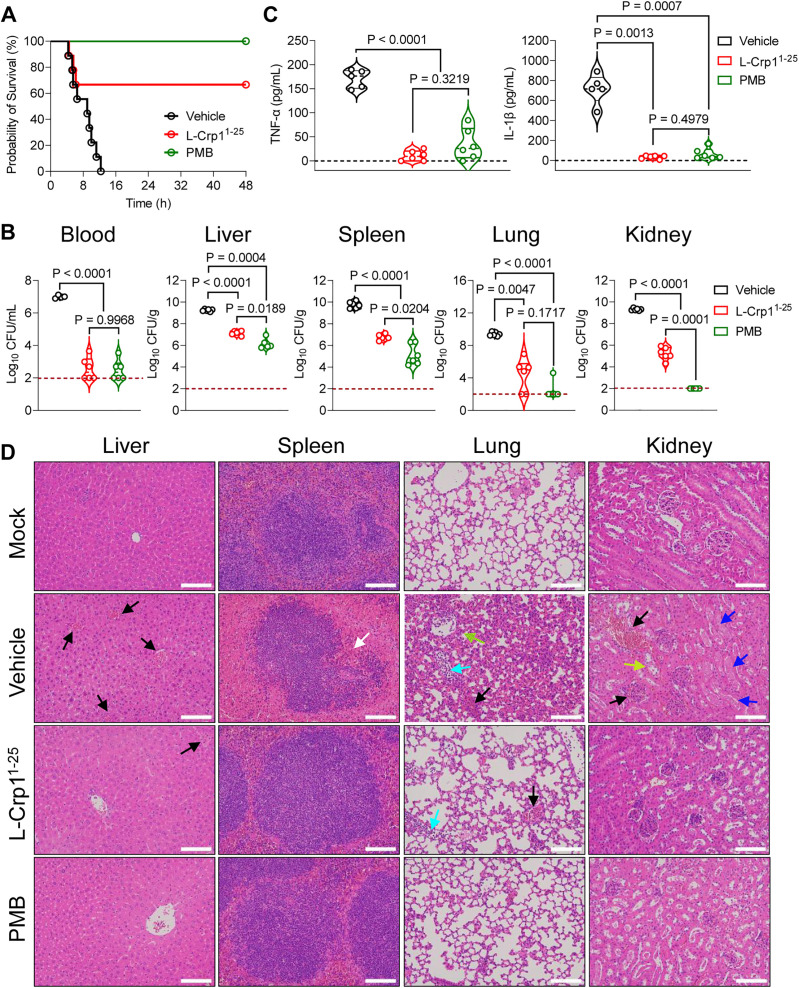
The truncated peptide L-Crp1^1-25^ rescues mice in a sepsis model. (A) Kaplan−Meier survival analysis of C57BL/6N mice (n = 9) were infected intraperitoneally with *E. coli* ATCC 25922 (5 × 10^7^ CFU/mice), followed by treatment with L-Crp1^1-25^ (20 mg/kg) or PMB (5 mg/kg). (B) Bacterial loads in the blood and organs of sepsis mice (n = 4 ~ 6). The red dashed line represents the detection limit (no colony growth on the agar plates). (C) Detection of cytokines IL-1β and TNF-α in mouse serum. Data are means ± SD. Statistical significance was determined using Brown-Forsythe and Welch ANOVA tests, followed by multiple comparisons between groups. (D) Representative histopathological images of H&E-stained sections of liver, spleen, lung and kidney sections. Black arrows, bleeding; white arrows, rupture of the red pulp and germinal center; cyan arrows, inflammatory infiltration; green arrows, exudation and alveolar membrane thickening; blue arrows, disruption of renal tubule and cell cast; yellow arrows, damaged brush border. Scale bar = 100 µm.

Despite that L-Crp1^1-25^ is slightly less active than L-Crp1 *in vitro*, its substantially smaller size (25 vs 35 amino acid residues) is advantageous in drug development. To evaluate the therapeutic efficacy of L-Crp1^1-25^, we intraperitoneally challenged male C57BL/6N mice with 5 × 10⁷ *E. coli* ATCC 25922 to establish a sepsis model, followed by treatment with L-Crp1^1-25^ or the positive control, polymyxin B (PMB) – the natural AMP clinically used as a last resort for the treatment of infections by multidrug-resistant Gram-negative bacteria [53]. The survival curves of mice in the infection group (vehicle), the treatment group (L-Crp1^1-25^), and the positive control group (PMB) over a 48-hour period are depicted in **Fig 7A**. While all mice in the infection group (n = 9) succumbed within 12 hours to bacterial infection, administration of two doses of PMB (5 mg/kg) at 0.5 and 2 hours post-infection resulted in 100% survival. Two doses of L-Crp1^1-25^ at 20 mg/kg administered at the same time intervals yielded a survival rate of 67%.

To further characterize the efficacy of L-Crp1^1-25^ in the sepsis model, murine blood, liver, spleen, lung, and kidney tissues were collected at 5 hours post-infection and processed for bacterial colony counting. As shown in Fig 7B, both L-Crp1^1-25^ and PMB significantly reduced bacterial burden, with blood bacterial counts dropping to the detection limit and organ bacterial counts decreasing by 2–4 orders of magnitude compared to the untreated infection group. Although L-Crp1^1-25^ treatment achieved a notable reduction in bacterial burden, PMB proved more effective than L-Crp1^1-25^, particularly in reducing bacterial load in the kidney, which may be attributed to a poor pharmacokinetics profile of the linear peptide.

Interestingly, despite being less effective than PMB in reducing bacterial burden, L-Crp1^1-25^ potently inhibited the release of the pro-inflammatory cytokines TNF-α and IL-1β into the blood (**Fig 7C**). In fact, sepsis-induced multi-organ injury was significantly diminished by treatment with either L-Crp1^1-25^ or PMB (**Fig 7D**). Without the treatment, however, bacterial challenge induced overt injury to multiple murine organs as evidenced by severe bleeding in the liver (black arrows), disruption of the red pulp and germinal center in the spleen (white arrows), extensive infiltration of inflammatory cells (cyan arrows), bleeding (black arrows), and large exudation as well as alveolar membrane thickening (green arrows) in the lung, and bleeding (black arrows), disruption of renal tubule (blue arrows) as well as disappearance of the brush border (yellow arrows) in the kidney (Fig 7D). By contrast, treatment with L-Crp1^1-25^ or PMB largely alleviated the above aspects of tissue damage in multiple organs as shown in Fig 7D, consistent with semi-quantitative histopathological scoring (S4 Fig).

Of note, L-Crp1^1-25^ showed little toxicity to HeLa, J774A.1 and red blood cells *in vitro*, contrasting the full-length peptide Crp1 (S5 Fig). Perhaps not surprisingly, PMB, known for its nephrotoxicity [54], was notably toxic to the macrophage-like cell line and slightly induced hemolysis of red blood cells at high concentrations in the toxicity assays. Thus, L-Crp1^1-25^ may be an attractive lead compound for further therapeutic development.

### Comparison of L-Crp1^1-25^ with other antimicrobial peptides

We previously used the vCC method to quantify the bactericidal activity of LL-37 [55], the extensively studied human cathelicidin peptide with a multiplicity of antimicrobial and immunomodulatory functions [56,57]. For comparison, we also determined the *E. coli*-killing activity of PL-5, a clinical stage α-helical peptide of 26 amino acid residues (Ac-KWKSFLKTFK SAAKTVLHTA LKAISS-NH_2_) developed as a topical spray for skin wound infections [58,59]. As demonstrated by the vLD values of these cationic antimicrobial peptides (S6A Fig), L-Crp1^1-25^, LL-37 and PL-5 were largely comparable in activity against *E. coli* 25922. While L-Crp1^1-25^ at 4.03 μg/ml or 1.36 μM (vLD99) reduced *E. coli* survival by two orders of magnitude, 3.4 μM of LL-37 and 0.62 μM of PL-5 were required to attain the same level of bactericidal activity. We also subjected these peptides to a cytotoxicity assay using HeLa cells as described earlier (S5 Fig) to gain additional insight into their therapeutic selectivity. As shown in S6B Fig, both PL-5 and LL-37 showed substantial cytotoxic effects on HeLa cells with CC50 values (defined as the peptide concentration at which cell viability is reduced by 50%) of 2.88 and 9.53 μM, respectively. By contrast, L-Crp1^1-25^ was largely non-cytotoxic at up to 100 μM – the highest concentration used, showcasing a favorable therapeutic window for L-Crp1^1-25^ in comparison with LL-37 and PL-5.

To further evaluate the suitability of L-Crp1^1-25^ as a potential lead compound for therapeutic development, we used LC-MS to measure its proteolytic stability in 2% mouse serum over a period of 24 h with PL-5 and LL-37 as controls. L-Crp1^1-25^ underwent a rapid cleavage at its C-terminal dibasic site -Arg^24^-Lys^25^, resulting in two major degradation products, i.e., an intermediate L-Crp1^1-24^ (M1) and a terminal L-Crp1^1-23^ (M2) as identified by mass spectrometry (S7A Fig). As expected, M1 initially increased over time and plateaued at 4 h, followed by a precipitous decrease to yield a progressively populated M2. At 24 h, L-Crp1^1-25^ was quantitatively converted to M2 with a half-life of merely 1.58 h. By contrast, PL-5 and LL-37 were significantly more resistant than L-Crp1^1-25^ to proteolytic degradation, with the former registering a half-life of 17.3 h and the latter largely unaffected by 2% serum (S7B-D Fig). Despite its susceptibility to serum, L-Crp1^1-25^ was fully stable in the presence of 150 mM NaCl, though (S7E Fig). These data pin-point a potential deficiency of L-Crp1^1-25^ as a therapeutic compound and afford an important glimpse into possible improvements for its therapeutic efficacy.

## Discussion

Disulfide bonding in mammalian defensins is fully conserved, with the Cys1-Cys6, Cys2-Cys4 and Cys3-Cys5 topology found in α-defensins and Cys1-Cys5, Cys2-Cys4 and Cys3-Cys6 in β-defensins [3,43,60]. Not surprisingly, disulfide bonding is critical for many of the functions of mammalian defensins [5,10], with a notable exception of the killing of Gram-negative bacteria [30–32]. The functional importance of disulfide bonding for defensins to kill Gram-negative bacteria is highly variable, ranging from deleterious, neutral, to essential [30–33,35,36], and yet, the molecular basis underlying this interesting phenomenon remains poorly understood. The current work is inspired by our previous finding that the mouse intestinal α-defensin Crp1 was among the weakest antimicrobial peptides out of 17 naturally occurring cryptdins studied, whereas its Cys-to-Ala variant, i.e., L-Crp1, became the most potent in the panel against *E. coli* [36]. Studying the molecular basis underlying the functional disparity between Crp1 and L-Crp1 is therefore important not only for better understanding the mechanisms of action of antimicrobial peptides in general, but also for designing more effective peptide antibiotics to treat drug-resistant bacterial infections.

We found that linearization of Crp1, a three-stranded β-sheet stabilized by three intramolecular disulfides, allowed L-Crp1 with an intrinsic α-helical propensity to adopt a drastically different helix-loop-helix conformation conducive to productive peptide-membrane interactions, leading to efficient bacteriolysis. This finding is further supported by the truncation of the C-terminal segment (GHLLYTLAAR) of L-Crp1, yielding L-Crp1^1-25^ with a similar structure and comparable bactericidal activity against Gram-negative bacteria. On the contrary, linearization of HNP4 yielded a significantly less active L-HNP4 that failed to adopt any helical conformation in the presence of TFE or SDS. Since cationic AMPs are generally believed to be able to efficiently traverse the highly anionic outer membrane [61–63], it is plausible that the functional outcome with respect to the killing of Gram-negative bacteria by a disulfide-devoid defensin peptide depends largely on its ability to adopt a helical conformation permissible to enhanced interactions with the microbial membrane.

We previously reported that certain cryptdins, including Crp1, had strong tendency to self-associate in solution, which was negatively correlated with their ability to kill *E. coli* [36]. The molecular basis for this negative correlation, however, remained obscure. Our SEM data revealed that Crp1, but not L-Crp1, readily formed nanonets cloaking *E. coli* cells or protein A beads, suggesting that the high-order assembly structurally precluded Crp1 from productively interacting with *E. coli* membrane. The accentuated ability of Crp1 to self-assemble into nanonets in the presence of *E. coli* or protein A beads also necessarily reduced its effective concentration to kill, likely explaining why L-Crp1 was much more potent than Crp1 against Gram-negative bacteria. These findings are reminiscent of an enteric α-defensin from humans, human α-defensin 6 or HD6. HD6 itself is weakly bactericidal [42] but can form nanonets in the intestine to entrap bacterial pathogens such as *Salmonella*, thus preventing them from breaching the host epithelium [64,65]. More recently, self-assembled HD6 has been shown to inhibit *Salmonella* mobility through binding to bacterial flagellin [66]. Of note, reduction or linearization of HD6 largely rescued its lost bactericidal activity [67,68] as was the case with the weakly bactericidal human β-defensin 1 [33], suggesting that the phenomenon with Crp1/L-Crp1 may be a lot more common than previously thought.

Why, then, does nature make disulfide-bridged Crp1 when its unstructured form is far more active against certain types of bacteria? Peptides and small proteins such as defensins, due to the lack of a sizable hydrophobic core, are invariably stabilized by disulfide bonds to confer proteolytic resistance that is often critical for biosynthesis, trafficking and secretion [4,5,69]. Under physiological conditions, a disulfide-stabilized defensin may be more resilient or robust than its unstructured linear form as the antibacterial activity of defensins is known to be heavily influenced by ions, salts, serum, et al [5,70–72]. Besides, defensins do not always have to kill to protect the host as is the case with HD6 [64]. Importantly, defensins are also immunomodulatory [2,5,10,11,60], which necessitates their productive interactions in a disulfide-stabilized conformation with various host factors such as receptors [73,74]. In fact, defensins lose much of their multifaceted functions without disulfide bonds [28,30–32], suggesting that a subset of infectious microbes is not the only intended target of some host defense peptides whose disulfide-devoid linear forms happen to be more active. Since loss of disulfide bonding does not always lead to enhanced killing of Gram-negative bacteria by defensins, it is conceivable that the conformational diversity and functional multiplicity afforded by disulfide reduction of defensins, which can occur under physiologically conditions [75,76], may simply be another example of the promiscuity and versatility of these genetically abundant and redundant innate factors.

It is important to note that there are two common but different technical approaches to studying the effect of disulfide bonding on the function of a defensin peptide, (1) disulfide reduction by a reducing agent such as dithiothreitol, β-mercaptoethanol or tris(2-carboxyethyl)phosphine, and (2) Cys-to-Ala mutation. In the first approach, the reducing agent and, sometimes, solubility-enhancing denaturants such as guanidinium hydrochloride or urea may be present in the assay to prevent intramolecular thiol-disulfide exchanges and the reverting of a fully reduced peptide to various oxidized forms. We have preferably applied the second approach to studies of HBD1, HBD2, HBD3, HNP1, HNP4, HD5 and some cryptdins where neither reducing agent nor denaturant needs to be introduced and the Cys-to-Ala mutation potentially alleviates a functionally indiscernible presence of free thiols in these peptides. Among the many defensins we studied, Crp1 was the only one whose (Cys-to-Ala) linearized form exhibited substantially enhanced bactericidal activity against Gram-negative bacteria. Of note, disulfide reduction of the weakly bactericidal human β-defensin HBD1 potentiates its antimicrobial activity against *Candida albicans* and anaerobic, Gram-positive commensals, a bactericidal effect dependent on the C-terminal free Cys residues [33]. Consistent with this finding, a Cys-to-Ala mutation in HBD1 failed to improve its bactericidal activity against *E. coli* and *S. aureus* (S2 Table). Obviously, enhanced killing of Gram-positive or -negative bacteria because of the loss of disulfide bonding in a defensin is neither a foregone conclusion nor a universal phenomenon.

L-Crp1^1-25^ displayed an efficacious anti-infective and anti-inflammatory activity in mice challenged with intraperitoneally inoculated *E. coli*. Despite its less active *in vivo* activity against *E. coli* infection than PMB, L-Crp1^1-25^ exhibited a superior safety profile, thus promising a potential lead compound for therapeutic development. Antibiotic resistance caused 4.95 million deaths worldwide in 2019 and this number is expected to rise to 8.22 million by 2050 [77]. Clinically, most antibiotic-resistant infections (>70%) are caused by Gram-negative bacteria such as *A. baumannii* and *E. coli*. It is thus of particular importance to develop new classes of anti-infective agents refractory to the existing resistance mechanisms by which Gram-negative bacteria evade conventional antibiotics [78]. Cationic antimicrobial peptides are attractive candidates as they primarily target the microbial membrane in a process where resistance is less likely to develop due to the relatively conserved nature of bacterial membranes [44,79]. In this regard, AMPs are advantageous over traditional antibiotics that often target a specific pathway or protein, which can readily evade through mutations.

Despite the promise, AMPs are faced with some pharmacological hurdles; more work is obviously needed to improve L-Crp1^1-25^’s antibacterial potency, proteolytic stability and pharmacokinetics for it to be a creditable drug candidate for therapeutic development. Future studies could be designed to interrogate the therapeutic efficacy of L-Crp1^1-25^ under more stringent experimental conditions such as delayed treatment, intravenous administration, and expanded cytokine survey. Of note, proteolytic excision of the dibasic residues at the C-terminus of L-Crp1^1-25^ reduces its cationicity and may also destabilize the helix-loop-helix structure as implied by MD simulations, thus negatively impacting its bactericidal activity. Since D-enantiomerization of defensins has no effect on their killing of Gram-negative bacteria because the microbial membrane target is achiral [30], an L-Crp1^1-25^ peptide composed entirely of D-amino acids, which is fully resistant to proteolysis, may be a perfect solution to this problem. Alternatively, various peptidomimetic chemistries can be devised to improve L-Crp1^1-25^’s pharmacological properties, including the use of sidechain stapling techniques to structurally stabilize α-helical peptides [80].

## Materials and methods

### Ethics statement

The animal study of mouse sepsis model was approved by the Ethics Committee of the School of Basic Medical Sciences at Fudan University (20230301–055).

### Peptides, bacterial strains and cell lines

Crp1 were synthesized by China Peptides (Shanghai) using Fmoc chemistry, then folded as previously described [36] in 50 mM Tris-HCl buffer (pH 8.3) containing 3 mM reduced glutathione, 0.3 mM oxidized glutathione, and 1 M guanidine hydrochloride, followed by purification using preparative reversed-phase high-pressure liquid chromatography (RP-HPLC). HNP4 and its linear form (L-HNP4) were laboratory stocks, and were synthesized, folded, and purified as described previously [35]. L-Crp1, L-Crp1^1-14^ and L-Crp1^1-25^ were synthesized by GL Biochem (China) using Fmoc chemistry. All peptides were verified on an Agilent HPLC Infinity II 1290 system coupled with a TOF 6230 detector prior to use.

The bacterial strains and the cell lines used in this study are listed in S1 Table. *E. coli* ATCC 25922, *K. pneumoniae* ATCC 13883, *P. aeruginosa* ATCC 27853 and *S. flexneri* 2a strain 301 were all cultured in LB broth at 37°C with 220-rpm agitation. HeLa and J774a.1 cells were cultured in DMEM medium containing 10% fetal bovine serum and 1% Pen-Strep (100 units/ml penicillium and 100 μg/ml streptomycin) in a humidified 5% CO_2_ atmosphere at 37°C.

### Antibacterial activity assay

The antibacterial activities of the peptides were evaluated by the “virtual colony count” assay, as previously reported [42]. Briefly, *E. coli* ATCC 25922, *K. pneumoniae* ATCC 13883, *P. aeruginosa* ATCC 27853 and *S. flexneri* 2a strain 301 [22,81] were cultured in LB broth to mid-logarithmic phase, then diluted to 1 × 10^6^ CFU/ml in 10 mM sodium phosphate buffer (pH 7.4). The bacterial suspensions were mixed with peptides at concentrations ranging from 1 to 256 μg/ml in a final volume of 100 μl, followed by incubation at 37°C for 2 hours. Next, 100 μl of 2 × Muller-Hinton broth (MHB) was added, and bacterial growth was monitored at 650 nm using a microplate reader (SpectraMax ABS, Molecular devices, USA) at 37°C for 12 hours.

### Bacterial viability assay

The viability of bacteria treated with Crp1 and other peptides was evaluated using the LIVE/DEAD BacLight bacterial viability kit (Invitrogen, Cat #: L7012). *E. coli* ATCC 25922 were grown to mid-logarithmic phase, washed with 10 mM sodium phosphate buffer, and diluted to 1 × 10^7^ CFU/ml. The peptides were then added to the bacterial suspensions to a final concentration of 4 μg/ml, with an equal volume of diluent added as a control. After incubation at 37°C for 1 hour, the bacteria were washed by and resuspended in 1 ml of 10 mM sodium phosphate buffer. Next, each sample was stained by adding 1.5 μl of SYTO9 (3.34 mM) and 1.5 μl of PI (20 mM). After incubation at room temperature in the dark for 15 min, fluorescent images of the stained bacteria were obtained using a confocal laser scanning microscope (Leica TCS SP8).

### Large unilamellar vesicles (LUVs) preparation and ANTS/DPX leak assay

The LUVs used in this study were prepared as previously described [28,47]. Briefly, 30 μmol of phospholipids (POPC:POPG = 1:1, 15 μmol each) were dissolved in 5 ml chloroform, and dried using a rotary evaporator to form a thin lipid film. The residual solvent was further removed under vacuum overnight. Subsequently, the dried lipid film was hydrated with 12.5 mM ANTS (Invitrogen, Cat #: A350), 45 mM DPX (Invitrogen, Cat #: X1525), 20 mM NaCl and 5 mM HEPES (Aladdin, Cat #: H109407) for 2 hours at 40°C. The lipid suspension was subjected to ten freeze–thaw cycles and extruded seven times through a 400nm polycarbonate filter using a Mini-Extruder (Avanti) at 40°C to produce LUVs. Unencapsulated dye was removed by gel filtration chromatography using a Sephadex G-50 column (Sigma, Cat #: G5050) with an eluent buffer containing 100 mM NaCl and 5 mM HEPES.

For the ANTS/DPX leak assay, ANTS/DPX-loaded LUVs (600 μM) were mixed with serially diluted peptides in a 96-well fluorescence assay plate (Greiner, Cat #: 655209). The 0.015% Triton X-100 solution was used as a positive control, while the eluent buffer served as a negative control. The plates were incubated for 2 hours at room temperature, and fluorescence was measured at an excitation wavelength of 350 nm and an emission wavelength of 420nm. The percentage of dye release was calculated using the following equation:


$$Percentage\;dye\;released(\%)=\frac{F-F_{\text{0}}}{F_{\text{1}\text{0}\text{0}}-F_{\text{0}}}\times \text{1}\text{0}\text{0}$$

where *F* is the fluorescence intensity in the presence of peptides, *F*_*0*_ is the fluorescence intensity of intact LUVs, and *F*_*100*_ is the fluorescence intensity after treatment with 0.015% Triton X-100.

### Circular dichroism (CD) spectrum

CD spectra were collected by a Jasco J - 725 spectrophotometer (Jasco) using a 1 mm pathlength quartz tube. The spectra were scanned under a nitrogen atmosphere, from 250 to 190 nm at a step of 0.1 nm, a scan speed of 50 nm/min, a response time of 1.0 s, and a scan bandwidth of 1.0 nm, with four scans averaged per time. Peptide samples (30 μM) were measured in 10 mM sodium phosphate buffer (pH 7.4), 40% trifluoroethanol (TFE), and 10 mM SDS.

The average residue ellipticity value *θ* was calculated using the following equation:


$$\theta =\frac{\theta_{observed}}{\text{1}\text{0}\times n\times C\times l}$$

where *θ*_*observed*_ is the measured ellipticity in degrees, *n* is the number of amino acid residues, *C* is the molar concentration of the peptide, and *l* is the cell pathlength in centimeters.

### Scanning electron microscopy

*E. coli* ATCC 25922 were cultured to mid-logarithmic phase, resuspended in 10 ml of 10 mM phosphate buffer (pH 7.4) at ~1 × 10^8^ CFU/ml, and incubated with 6 μg/ml peptides at 37°C for 2 hours. Then, the bacteria were pelleted, resuspended in Karnovsky’s fixative (2% paraformaldehyde, 2.5% glutaraldehyde in 0.06 M Sorensen’s phosphate buffer [0.2 M sodium phosphate, pH 7.2]), and fixed overnight at room temperature. Next, the bacteria were washed by 0.1 M phosphate buffer (pH 7.4) for three times, post-fixed in 1% osmium tetroxide for 2 hours in the dark, dehydrated through a graded ethanol series (30%, 50%, 70%, 80%, 90%, 95%, 100%, and 100%, 15 minutes each), and dried in a critical point dryer (Quorum, K850). The dried samples were placed on the adhesive and conductive carbon film and sputter-coated with gold for 30 seconds in an ion sputtering instrument (HITACHI, MC1000). Samples were photographed using a HITACHI SU8100 scanning electron microscope at 20 kV. The SEM characterization results were supported by Zhongkebaice Technology Service Co., Ltd. (Beijing, China) and Baiqiandu Biotechnology Co., Ltd. (Wuhan, China).

To exclude the influence of bacterial secretions, protein A-coated polystyrene beads (0.1% w/v, Spherotech, PAP-08–5) were treated with 6 μg/ml peptides for 30 minutes in 10mM phosphate buffer (pH 7.4), and then resuspended Karnovsky’s fixative and fixed overnight at room temperature. These beads were subsequently fixed, dehydrated, dried, sputter-coated, and photographed as described above.

### Surface plasmon resonance analysis

SPR measurements were performed using a BIACORE T200 system (GE Healthcare) at 25°C in 1 × HBS-EP+ buffer (10 mM HEPES, 150 mM NaCl, 3 mM EDTA, and 0.005% surfactant P20, pH 7.4, Cytiva). L-Crp1 (immobilized at 969.6 RUs) or Crp1 (1839.2 RUs) were immobilized on a CM5 sensor chip according to the manufacturer’s instructions. Indicated concentrations (3.9 to 1000 nM) of analytes were injected into the channels at 30 μl/min in the running buffer. The association and dissociation kinetics were monitored for 120 and 180 seconds, respectively. Resonance signals were corrected by subtracting the response of the blank control channel. The sensor chip was regenerated with 10 mM glycine (pH 1.5) and re-equilibrated with the running buffer after each analysis.

### Dynamic light scattering analysis

Dynamic light scattering measurements were performed using a Malvern Zetasizer Nano (Malvern Panalytical). Crp1 and its linearized analog (L-Crp1) were dissolved in 10 mM phosphate buffer (pH 7.4) at the concentration of 10 μg/ml, and filtered through a 0.22-μm PES membrane. After equilibrating for 120 seconds at room temperature, the relative intensity of scattered light was measured. Since the scattering intensity is highly sensitive to particle size, resulting in an intensity distribution that is strongly skewed toward larger particles, we present the number distributions data that converted from intensity distributions through Mie theory using the manufacturer’s software (Zetasizer Software 7.12).

### *De novo* folding of L-Crp1 by molecular dynamics (MD) simulation

Three-dimensional structure of Crp1 was modelled by AlphaFold using default parameters [82], and the initial structure of L-Crp1 was constructed using PyMOL by mutating Cys to Ala. L-Crp1 were assigned molecular mechanic parameters from ff19SB force field [83], and were further solvated by in a periodic boundary cuboid box of transferable interatomic potential with three points model (TIP3P) water molecules with solvent layers 12.0 Å between solute surface and box edges, via the LEaP module of Amber20. Na^+^/Cl^+^ were employed as counters to neutralize peptides with addition of 10 mM NaCl to the TIP3P box.

MD simulation of *de novo* folding of L-Crp1 was conducted on Amber20 with SHAKE algorithm employed to constrain all covalent bonds involving hydrogen atoms. The simulated system was initially energetically minimized by applying a 10000-step steepest descent minimization and a 10000-step conjugate gradient minimization, respectively, with heavy atoms restrained by 25 kcal mol^-1^Å^-2^, ensuring the water molecules distributed uniformly. The simulated system was subsequently undergone a 5000-step steepest descent minimization and a 5000-step conjugate gradient minimization, with C_α_ atom restrained by 25, 10, and 5 kcal mol^-1^Å^-2^, respectively. Finally, the system was freely relaxed by 10,000 steps of steepest descent minimization and 10,000 steps of conjugate gradient minimization without any constraints, which was then heated to 300 K in a time step of 2 fs under condition of constant volume (NVT) with heavy atoms restrained by 5 kcal mol^-1^Å^-2^. A 200-ps density simulation under condition of constant pressure (NPT) was applied to the heated system that was subsequently undergone two steps of NPT equilibrium with or without restraining of heavy atoms. The simulated system was finally submitted to a 100-ns NPT production simulation (T = 300 K, P = 1 atm) using isotropic pressure scaling on GPU platform of NVIDIA GeForce RTX 3080 Ti.

Trajectory was analyzed by CPPTRAJ, and Gibbs free energy alterations (ΔG) were calculated using MM/GBSA method [84]. All molecular structures were visualized using PyMOL (v2.0, open-source), and the data were plotted using GraphPad Prism 8.

### MD simulations of interaction between Crp1/L-Crp1 and bacterial membrane

To study mobility of peptides approaching bacterial inner membrane in solution, we initially constructed a lipidic bilayer by a lipid ratio of POPG:POPC = 1:3 to mimic bacterial inner membrane, using CHARMM-GUI web server (https://www.charmm-gui.org/) [85]. Crp1 or L-Crp1 were randomly put onto the outer leaflet of bilayer using Packmol module [86], which were solvated by in a periodic boundary cuboid box of TIP3P water molecules with solvent layers 12.0 Å between solute surface and box edges, via the LEaP module of Amber20. And the molecular mechanics parameters from ff19SB and lipid14 force fields were assigned to above solutes [83]. The simulated system was then neutralized by adding Na^+^/Cl^+^ as counters, and additionally introduced 150 mM NaCl to mimic physiological salt conditions. To extensively probe the binding strength of Crp1/L-Crp1 with bilayer, GHARMM-GUI web server was employed to construct bacterial bilayer with lipid ratio of POPG:POPC = 1:3 with peptide molecules fixed onto and slightly contacted with the outer leaflet of the bilayer using the same Z-axis distance. Conditions of force fields and salt were used as above settings.

MD simulation was conducted on Amber20 with SHAKE algorithm employed to constrain all covalent bonds involving hydrogen atoms. The simulated system was initially energetically minimized by applying a 10000-step steepest descent minimization and a 10000-step conjugate gradient minimization, respectively, with heavy atoms restrained by 25 kcal mol^-1^Å^-2^, ensuring the water molecules distributed uniformly. The simulated system was subsequently undergone a 5000-step steepest descent minimization and a 5000-step conjugate gradient minimization, with C_α_ atom restrained by 25, 10, and 5 kcal mol^-1^Å^-2^, respectively. Finally, the system was freely relaxed by 10,000 steps of steepest descent minimization and 10,000 steps of conjugate gradient minimization without any constraints. To avoid severe fluctuation resulted from heating, the simulated system was slowly heated to 100 K under NVT conditions with a time step of 0.5 fs, followed by heating from 100 K to 300 K with a time step of 2 fs with heavy atoms restrained by 5 kcal mol-1Å-2. A 200-ps NPT density equilibration was applied to the heated system that was subsequently undergone two steps of NPT equilibration with or without restraining of heavy atoms. The simulated system was finally submitted to a 100-ns or 200-ns NPT production simulation (T = 300 K, P = 1 atm) using semi-isotropic pressure scaling on GPU platform of NVIDIA GeForce RTX 3080 Ti. Trajectories and molecular structures were analyzed and visualized as described above.

### Mouse sepsis model

The animal study was approved by the Ethics Committee of the School of Basic Medical Sciences at Fudan University (20230301–055). All animal experimental procedures strictly adhered to the International Ethical Guidelines and the National Institutes of Health Guide concerning the Care and Use of Laboratory Animals. In this study, specific-pathogen-free male C57BL/6N mice (Charles River, China), aged 8 weeks and weighing 23 ± 2 g, were used.

To prepare the bacteria, *E. coli* ATCC 25922 was cultured in LB overnight at 37°C and 220 rpm, and then 1:100 subcultured to the mid-log phase the next day. The bacteria were collected by centrifugation (4000 rpm, 5 minutes, room temperature), washed once with 10 mM phosphate buffer, and resuspended in 10 mM phosphate buffer to the concentration of 2.5 × 10^8^ CFU/ml.

As for infection, each mouse was intraperitoneally injected with 200 μl of the resuspended bacteria (~5 × 10^7^ CFU per mouse). Then the mice were treated by intraperitoneal injection of L-Crp^1-25^ (20 mg/kg) 0.5 and 2 hours post bacterial challenge. The positive controls were treated by 5 mg/kg PMB. The mouse survival was monitored for 48 hours post-infection (n = 7).

In addition to the survival study, another experiment with the same experimental settings (n = 6) was also performed to further characterize the sepsis model. Five hours after bacterial infection, the mice were anesthetized, and blood and organs (liver, spleen, lungs, and kidneys) were collected. Half of each organ was weighed, homogenized, and diluted in PBS for bacterial colony counting, while the remaining tissue was fixed in 4% paraformaldehyde, embedded in paraffin, and sectioned for H&E staining. The H&E stain and imaging were supported by Pinuofei Biological Technology company (Wuhan, China). Organ damage was assessed by examining three randomly selected fields from each sample at a 200 × magnification. Histopathological scoring was conducted according to our previous publication [87]. For bacterial load assessment, the blood and the diluted tissue suspensions were plated on LB agar plates and incubated at 37°C for 12 hours. The bacterial loads in the tissues were expressed as lg(CFU/g), while those in the blood were expressed as lg(CFU/mL). Two inflammatory cytokines in serum, *e.g.,* TNF-α and IL-1β, were measured using the ELISA kits (ProteinTech, catalog no. KE10002 and KE10003) according to the manufacturer’s instructions.

### Cell cytotoxicity assay

HeLa and J774A.1 cells were seeded into 96-well plates (~3 × 10⁴ cells/well) respectively, and cultured for 16–20 hours. Then, the medium was replaced with 100 µl/well of DMEM containing peptides of indicated concentrations (1–256 µg/ml). Next, the cells were incubated for another 24 hours. To detect the cell viability, 10 µl/well of the CCK-8 solution (MCE, China) was added to the plates. After 1 hour of incubation, the cell viability was measured by reading the absorbance at 450 nm using a microplate reader (SpectraMax ABS, Molecular devices, USA).

### Peptide stability assays

Tested peptides including L-Crp1^1-25^, PL-5 or LL-37 were diluted in 10 mM PB buffer containing 2% mouse serum (or, separately, 150 mM NaCl in the case of L-Crp1^1-25^), at a final peptide concentration of 400 μg/mL, followed by incubation at room temperature for various time lengths. 50 μL of peptide solution was withdrawn at indicated time points (0, 0.5, 1, 2, 4, 6, 8, and 24 h) and then mixed with an equal volume of ice-cold acetonitrile, followed by HPLC-TOF-MS detection and quantification at 40 °C on an Agilent AdvanceBio Peptide Plus Column (2.7 μm, 2.1 × 150 mm) running a solvent gradient over 15 min at a flow rate of 0.4 mL/min (mobile phase A: 0.1% TFA in H_2_O, mobile phase B: 0.1% TFA in acetonitrile). The gradient was 10–20% B for L-Crp1^1-25^ and 30–45% B for PL-5 and LL-37.

## Supporting information

S1 Fig HPLC-TOF-MS analysis of Crp1 and L-Crp1. **(A)** The HPLC chromatogram of Crp1 and its ESI-TOF mass spectrometric data. The chromatogram was obtained at 60 °C on an Agilent 300 SB-C18 Column (1.8 μm, 2.1 × 50 mm) running a solvent gradient of 5–65% B over 5 min at a flow rate of 0.2 mL/min (mobile phase A: 0.1% TFA in H_2_O, mobile phase B: 0.1% TFA in acetonitrile). **(B)** Deconvolution of the mass spectrum yields an observed molecular mass of 4116.05 Da, in agreement with the theoretical value of 4115.88 Da calculated according to the average isotopic compositions of Crp1. **(C)** The HPLC chromatogram of L-Crp1 and its ESI-TOF mass spectrometric data obtained under the same conditions as described for Crp1. **(D)** Deconvolution of the mass spectrum yields an observed molecular mass of 3929.25 Da, in agreement with the theoretical value of 3929.57 Da calculated according to the average isotopic compositions of L-Crp1.
(TIF)

S2 Fig SEM images of *E. coli* ATCC 25922 or protein A polystyrene beads treated with HNP4 or L-HNP4. The nanonet structures in the L-HNP4-treated groups are indicated with white arrows. Scale bar = 1 μm.
(TIF)

S3 Fig Particle size distributions of HNP4 and L-HNP4 analyzed by DLS. (S3_Fig.TIF)

S4 Fig Severity of organ injury in the liver, spleen, lung, and kidney as quantified by histopathological scoring. n = 5 ~ 6 per group. Data are presented as mean ± SD. Statistical significance was calculated by two-way analysis of variance with the Bonferroni correction for multiple comparisons. *P < 0.05, **P < 0.01, ***P < 0.001, ****P < 0.0001.
(TIF)

S5 Fig *In vitro* cytotoxicity and hemolytic activity of the indicated peptides. **(A)** Cytotoxicity of Crp1, L-Crp1, L-Crp1^1-25^ and PMB to HeLa (left) and J774.A1 (right) cells as evaluated by the CCK-8 assay. Results are mean ± SD of data from two independent experiments. **(B)** Hemolytic activity of the peptides on red blood cells (RBC) from C57BL/6N mice. Data are shown as mean ± SD from assays in two replicates.
(TIF)

S6 Fig Comparison of L-Crp1^1-25^, LL-37 and PL-5 with respect to bactericidal activity against *E. coli* and cytotoxicity to HeLa cells. **(A)** Virtual lethal doses (vLD) of L-Crp1^1-25^, LL-37 and PL-5 against *E. coli* ATCC29522 as determined by the virtual colony count assay. *Data of LL-37 cited from a previous publication (*Pazgier M et al., Biochemistry 2013*). **(B)** Cytotoxicity of L-Crp1^1-25^, LL-37 and PL-5 to HeLa cells as evaluated by the CCK-8 assay. Results are mean ± SD of data from assays in triplicate.
(TIF)

S7 Fig Characterization of peptide stability in the presence of 2% mouse serum or 150 mM NaCl by HPLC-TOF-MS. Shown in the left panels are HPLC (A-C and E) or mass spectrometric (D) traces of the peptides following incubation with serum (A-D) or salt (E) for different time lengths, and in the right panel are their time-dependent degradation as quantified by integration of chromatographic or mass spectrometric peaks. **(A)** L-Crp1^1-25^ with serum by chromatography, **(B)** PL-5 with serum by chromatography, **(C)** LL-37 with serum by chromatography, **(D)** LL-37 with serum by mass spectrometry, **(E)** L-Crp1^1-25^ with salt by chromatography. M1 (LRDLVAYARS^10^ RGAKGRERMN^20^ GTAR) and M2 (LRDLVAYARS^10^ RGAKGRERMN^20^ GTA) are two major degradation products of L-Crp1^1-25^. The extracted-ion chromatograms (EIC) **(D**, left) for 1124 ± 1 m/z were used to quantify LL-37 levels **(D**, right) due to coelution of LL-37 with a serum protein **(C)**.
(TIF)

S1 Table Bacterial strains and cell lines used in this study. (DOCX)

S2 Table Antibacterial virtual lethal doses of HBD1, Crp1, L-Crp1, L-Crp1^15-25^, and L-HBD1 against *E. coli* ATCC 25922 and *S. aureus* ATCC 25923. The virtual lethal doses (vLD, μg/mL) of peptides required to kill 50%, 90%, 99%, and 99.9% of viable cells from various bacteria inputs were determined. These assays were performed triplicate.
(DOCX)

S1 Data Raw data used to generate article figures. (XLSX)
