# Supplementary material for: Breaking disulfide bonds in a weakly bactericidal α-defensin unleashes a potent antimicrobial peptide with an altered conformation
Source: PLoS Pathog. 2026 Feb 9;22(2):e1013954. doi: 10.1371/journal.ppat.1013954 (PMC12904582; doi:10.1371/journal.ppat.1013954)
Supplement: S1 Table — (DOCX) [file ppat.1013954.s008.docx]

**S1 Table. Bacterial strains and cell lines used in this study.**

| **Bacterial strains and cell lines** | **Source** |
| --- | --- |
| **Bacterial strains** |  |
| *Escherichia coli* ATCC 25922 | Lab stock (ATCC 25922) |
| *Klebsiella pneumoniae* ATCC 13883 | Lab stock (ATCC 13883) |
| *Pseudomonas aeruginosa* ATCC 27853 | Lab stock (ATCC 27853) |
| *Shigella flexneri* 2a strain 301 | Lab stock |
| **Cell lines** |  |
| HeLa | Lab stock (ATCC CCL-2) |
| J774.A1 | Lab stock (ATCC TIB-67) |
