# Supplementary material for: Breaking disulfide bonds in a weakly bactericidal α-defensin unleashes a potent antimicrobial peptide with an altered conformation
Source: PLoS Pathog. 2026 Feb 9;22(2):e1013954. doi: 10.1371/journal.ppat.1013954 (PMC12904582; doi:10.1371/journal.ppat.1013954)
Supplement: S2 Table — The virtual lethal doses (vLD, μg/mL) of peptides required to kill 50%, 90%, 99%, and 99.9% of viable cells from various bacteria inputs were determined. These assays were performed triplicate. (DOCX) [file ppat.1013954.s009.docx]

**S2 Table. Antibacterial virtual lethal doses of HBD1, Crp1, L-Crp1, L-Crp1^15-25^, and L-HBD1 against *E. coli* ATCC 25922 and *S. aureus* ATCC 25923.** The virtual lethal doses (vLD, μg/mL) of peptides required to kill 50%, 90%, 99%, and 99.9% of viable cells from various bacteria inputs were determined. These assays were performed triplicate.

| **Bacteria** | **Peptides** | **vLD50** | **vLD90** | **vLD99** | **vLD99.9** |
| --- | --- | --- | --- | --- | --- |
| ***E. coli***  **ATCC 25922** | HBD1 | 109±83.9 | >256 | >256 | >256 |
|  | Crp1 | 5.30±3.19 | 9.96±2.54 | 16.4±1.73 | 21.7±1.21 |
|  | L-Crp1 | 2.02±0.01 | 2.29±0.18 | 3.05±0.24 | >4 |
|  | L-Crp1^15-25^ | >256 | >256 | >256 | >256 |
|  | L-HBD1 | >256 | >256 | >256 | >256 |
| ***S. aureus***  **ATCC 25923** | HBD1 | >256 | >256 | >256 | >256 |
|  | Crp1 | 3.71±1.46 | 6.43±2.26 | >64 | >64 |
|  | L-Crp1 | 3.28±0.82 | 9.57±3.28 | 23.9±0.98 | >32 |
|  | L-Crp1^15-25^ | >256 | >256 | >256 | >256 |
|  | L-HBD1 | >256 | >256 | >256 | >256 |
